# An Examination of the Effect of Aspirin and Salicylic Acid on Soluble Fms-like Tyrosine Kinase-1 Release from Human Placental Trophoblasts

**DOI:** 10.3390/cells13020113

**Published:** 2024-01-06

**Authors:** Jiawu Zhao, Rui Duan, Jinghui Sun, Rebecca P. Chow, Timothy J. Lyons, Jeremy Y. Yu

**Affiliations:** 1 Wellcome-Wolfson Institute for Experimental Medicine, School of Medicine, Dentistry and Biomedical Sciences, Queen’s University Belfast, Belfast BT9 7BL, UK; zjwandst@yahoo.co.uk (J.Z.); pyrc201@yahoo.com (R.P.C.); lyonstj@musc.edu (T.J.L.); 2 Division of Endocrinology, Diabetes and Metabolic Diseases, Department of Medicine, Medical University of South Carolina, 96 Jonathan Lucas Street, Charleston, SC 29425, USA; ruiduan511@163.com (R.D.); sunji@musc.edu (J.S.); 3 Diabetes Free South Carolina, BlueCross BlueShield of South Carolina, Columbia, SC 29229, USA

**Keywords:** aspirin, hypoxia, preeclampsia, salicylic acid, soluble fms-like tyrosine kinase-1, trophoblast

## Abstract

Low-dose aspirin (LDA) is efficacious in preventing preeclampsia, but its mechanism of action is unclear. Conflicting evidence suggests that it may inhibit placental trophoblast release of soluble fms-like tyrosine kinase-1 (sFlt1), a key mediator of preeclampsia. We examined whether, and at what concentrations, aspirin and its principal metabolite, salicylic acid, modulate sFlt1 release and/or expression in trophoblasts. Human trophoblast lines BeWo and HTR-8/SVneo were cultured; BeWo cells were also treated with 1% oxygen vs. normoxia to mimic hypoxia in preeclamptic placentas. Cells were treated with aspirin or salicylic acid vs. vehicle for 24 h at concentrations relevant to LDA and at higher concentrations. Protein concentrations (ELISA) and mRNA expression (RT-PCR) of sFlt1 were determined. Under normoxia, LDA-relevant concentrations of aspirin (10–50 µmol/L) or salicylic acid (20–100 µmol/L) had no significant effect on sFlt1 protein release or mRNA expression in BeWo cells. However, inhibition was observed at higher concentrations (1 mmol/L for aspirin and ≥200 μmol/L for salicylic acid). Hypoxia enhanced sFlt1 protein release and mRNA expression in BeWo cells, but these responses were not significantly affected by either aspirin or salicylic acid at LDA concentrations. Similarly, neither drug altered sFlt1 protein secretion or mRNA expression in normoxic HTR-8/SVneo cells at LDA concentrations. We suggest that direct modulation of trophoblast release or expression of sFlt1 is unlikely to be a mechanism underlying the clinical efficacy of LDA in preeclampsia.

## 1. Introduction

Preeclampsia is a serious complication of pregnancy that affects 3–5% of women in the general population and ~20% of those with high-risk conditions, including diabetes [1,2]. Preeclampsia is characterized as new-onset hypertension during the second half of pregnancy in a previously normotensive woman, accompanied by proteinuria and/or other end-organ damage [3]. It is thought to originate from the under-development of uterine spiral arteries early in pregnancy, later resulting in an ischemic and hypoxic placenta that releases ‘toxic’ circulating factors into the maternal circulation, eventually causing vascular endothelial damage [4]. A key circulating factor that has been implicated is soluble fms-like tyrosine kinase-1 (sFlt1), an alternatively spliced extracellular fragment of vascular endothelial growth factor (VEGF) receptor 1 [5,6]. Excessive levels of sFlt1 scavenge VEGF and placental growth factor (PlGF), causing a deficiency of angiogenic signaling that, in turn, leads to endothelial dysfunction and vasoconstriction. Plasma levels of sFlt1 are elevated weeks before clinical onset of preeclampsia [2,5,6] and correlate with disease severity [7]. Accumulating preclinical [8,9] and clinical evidence [10,11] supports the notion of sFlt1 as a potential target for therapeutic development.

Current treatment options for preeclampsia are limited, mainly comprising symptomatic management of hypertension and seizures. The only medication that has shown some disease-modifying effect is aspirin (acetylsalicylic acid). Since 1978, numerous clinical studies have demonstrated the beneficial role of low-dose aspirin (LDA) in preventing preeclampsia [12]. Systematic reviews of randomized clinical trials found this effect to be modest overall (i.e., 10–18% efficacy) [13,14,15], and currently, a daily dosage of 81 mg is recommended for high-risk pregnancies [16,17]. Interestingly, the recent Aspirin for Evidence-Based Preeclampsia Prevention (ASPRE) trial showed that 150 mg daily reduced the incidence of preterm preeclampsia by 62%, suggesting a dose-dependent effect [12,18]. However, the underlying mechanism of action remains elusive. Some authors have suggested that aspirin might inhibit sFlt1 release from placental trophoblasts [19,20,21,22], while others did not find such an effect [23,24]; however, in some of these earlier studies, aspirin was studied at much higher concentrations than are practically achievable in pregnant women. Furthermore, earlier work focused on aspirin only and did not explore the possible role of its principal metabolite, salicylic acid; the latter is known to reach higher plasma concentrations than the parent drug and to mediate at least some of aspirin’s pharmacologic actions.

To date, available randomized clinical trials have not documented a reduction of plasma sFlt1 by LDA in pregnant women [25,26,27]; therefore, although plausible, this putative mechanism of action merits careful examination, considering that sFlt1 plays a critical role in connecting placental pathologies to maternal symptoms. If confirmed, aspirin’s effect on sFlt1 might be exploitable for the development of next-generation therapies with better potency and/or efficacy.

In the present study, we determined whether aspirin and/or its metabolite salicylic acid could modulate sFlt1 protein release and/or mRNA expression of either of the two predominant transcripts (*i13* and *e15a*) in cultured human trophoblasts and whether they do so at clinically relevant concentrations. The choriocarcinoma-derived cell line BeWo is a main model for villous syncytiotrophoblasts [28], the predominant placental source of sFlt1 [29]. The non-tumor cell line HTR-8/SVneo is frequently used to simulate cytotrophoblasts [30]. Since we previously found that hypoxia stimulated sFlt1 release in BeWo cells (but not in HTR-8/SVneo cells) [31], we also evaluated drug effects in this cell line under hypoxic conditions.

## 2. Materials and Methods

### 2.1. Cell Culture

Human placental trophoblast BeWo cells (ATCC, Manassas, VA, USA) were maintained in DMEM/F12 (Gibco, Thermo Fisher, Waltham, MA, USA) supplemented with 2 mM L-glutamate, and HTR-8/SVneo trophoblasts (a gift from Professor Charles Graham, Queen’s University at Kingston, ON, Canada) were cultured in RPMI 1640 (Sigma-Aldrich, St. Louis, MO, USA). All growth media contained 10% fetal calf serum and were devoid of detectable levels of sFlt1.

Prior to drug treatments, BeWo and HTR-8/SVneo cells were harvested by trypsinization and then seeded onto 48-well culture plates at 6 × 10^4^ and 1.2 × 10^5^ cells, respectively, to reach 80–90% confluence in approximately 24 h. For the BeWo-hypoxia model, cells were seeded onto the 48-well culture plate overnight, pre-treated with the drugs for 1 h, and then subjected to ambient oxygen vs. 1% oxygen (with 5% CO_2_ and 94% N_2_) in a humidified, temperature-controlled hypoxia chamber (Coy Laboratories, Grass Lake, MI, USA) for 24 h. The supernatants and cell pellets were collected for protein and mRNA expression assays, as described below. Cultured cells were inspected microscopically and confirmed free of discernible morphological changes or death.

### 2.2. Drug Materials

Aspirin and salicylic acid were purchased from Sigma-Aldrich (St. Louis, MO, USA). Chetomin was obtained from Santa Cruz Biotechnology (Dallas, TX, USA). Fresh drug solutions were prepared in ethanol per manufacturer’s instructions on the day of experiments and spiked into cell culture media to achieve the final test concentrations. The vehicle itself had no effect on cellular expression or release of sFlt1.

### 2.3. LDA-Relevant Drug Concentrations

In order to determine the test drug concentrations for cell culture studies, we surveyed the available clinical pharmacokinetic data for the LDA dose range of 40–150 mg/day. As summarized in Table 1, there were dose-related increases in plasma concentrations of both aspirin and salicylic acid, with the latter substantially higher than the former (the salicylic acid/aspirin maximum concentration (Cmax) ratio ranged from 2.0 to 18.1). While most published data were from healthy, non-pregnant male and female volunteers, at least two studies have shown that plasma Cmax and area under the curve (AUC) drug exposures were lower in pregnant than non-pregnant women [32,33]. Overall, the Cmax concentrations for aspirin were in the range of single-digit μmol/L for the 40–100 mg doses (and that for the 150 mg dose was not significantly higher as estimated from the salicylic acid data [33]), except for one study that showed an aberrantly higher value of 23.4 μmol/L [34]. For salicylic acid, the Cmax values ranged from 2.3–34.7 μmol/L for the 40–150 mg dose range, again with the exception of one study showing 57.4 μmol/L [34]. Considering that Cmax occurs transiently and the average concentration over time is much lower, we chose approximate concentrations of aspirin (10 and 50 μmol/L) and salicylic acid (20 and 100 μmol/L) to represent LDA in pregnant women in our experiments. Although plasma concentrations of aspirin close to or in the millimolar range are typically not experienced by pregnant women, they have been used in earlier cell culture studies; we, therefore, included concentrations of up to 1000 μmol/L aspirin and 2000 μmol/L salicylic acid in some experiments for comparison.

### 2.4. Enzyme-Linked Immunosorbent Assay (ELISA)

Cell culture supernatants of different treatment conditions were collected and clarified at 2000 g (10 min, 4 °C) to remove cellular debris. Protein concentrations of sFlt1 were quantified in duplicate using the Quantikine ELISA kit (R&D Systems, Minneapolis, MN, USA) according to the manufacturer’s instructions.

### 2.5. Real-Time Quantitative PCR (RT-PCR)

Total RNA was isolated from the cell pellets by the RNeasy mini kit (Qiagen, Hilden, Germany). The RNA concentration was assessed by the NanoDrop 1000 spectrophotometer, and 1 μg RNA was reverse-transcribed into cDNA by the First-Strand cDNA synthesis kit (Thermo Fisher, Waltham, MA, USA) following manufacturer’s protocol. The level of mRNA expression was quantified by RT-PCR using the LightCycler 480 system with SYBR Green I Master (Roche Diagnostics GmbH, Mannheim, Germany). The primers included *sFlt1 i13* forward: ACAATCAGAGGTGAGCACTGCAA, *sFlt1 i13* reverse: TCCGAGCCTGAAAGTTAGCAA; *sFlt1 e15a* forward: ACACAGTGGCCATCAGCAGTT, *sFlt1 e15a* reverse: CCCGGCCATTTGTTATTGTTA; *β-actin* forward: TGGGACGACATGGAGAAAAT, *β-actin* reverse: GAGGCGTACAGGGATAGCAC. *β-Actin* was used as a housing-keeping gene. Gene expression fold changes were calculated by dividing the normalized values of control samples by normalized values of treated samples.

### 2.6. Data Analysis

For comparison of normoxic and hypoxic conditions in BeWo cells, unpaired two-tailed Student’s *t*-tests were used. Drug treatment effects were analyzed by one-way ANOVA followed by Dunnett’s multiple comparison tests (Prism 8, GraphPad Software, San Diego, CA, USA). Drug exposure data were expressed as means ± SD, and efficacy data were presented as mean ± SE. *p* values < 0.05 were considered statistically significant.

## 3. Results

### 3.1. Effects of Aspirin and Salicylic Acid on sFlt1 Protein Release and mRNA Expression in BeWo Cells

**Figure 1:**
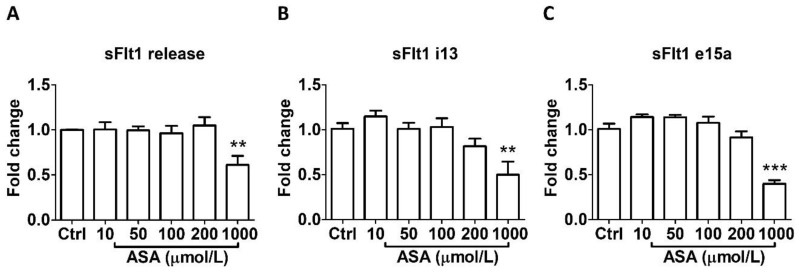
Concentration-dependent effects of aspirin (10–1000 μmol/L, 24 h) on sFlt1 protein release and mRNA expression in BeWo cells. (**A**) Effects of aspirin on supernatant sFlt1 protein concentrations. (**B**,**C**) Effects of aspirin on *sFlt1 i13* and *e15a* mRNA expression. ASA, aspirin. Data are expressed as fold changes relative to the control and presented as means ± SE, *n* = 6. ** *p* < 0.01 and *** *p* < 0.001.

As shown in Figure 1, aspirin treatment at LDA concentrations (10–50 μmol/L) for 24 h did not affect sFlt1 protein release (Figure 1A) nor mRNA expression (Figure 1B,C) in BeWo cells. However, 1000 µmol/L aspirin significantly reduced sFlt1 protein concentrations (by 39%, Figure 1A); it also significantly inhibited both *sFlt1 i13* (Figure 1B) and *e15a* (Figure 1C) mRNA expression.

**Figure 2:**
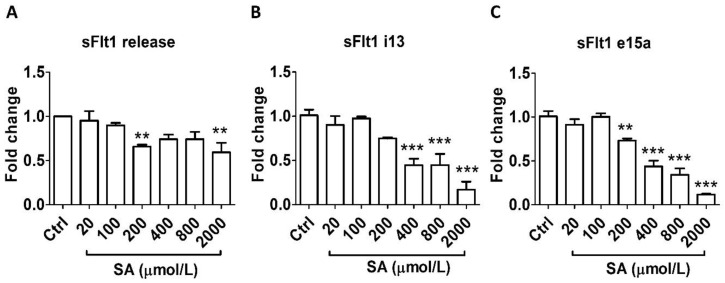
Concentration-dependent effects of salicylic acid (20–2000 μmol/L, 24 h) on sFlt1 protein release and mRNA expression in BeWo cells. (**A**) Effects of salicylic acid on supernatant sFlt1 protein concentrations. (**B**,**C**) Effects of salicylic acid on *sFlt1 i13* and *e15a* mRNA expression. SA, salicylic acid. Data are expressed as fold changes relative to the control and presented as means ± SE, *n* = 6. ** *p* < 0.01 and *** *p* < 0.001.

Similarly, salicylic acid did not alter sFlt1 protein secretion at concentrations relevant to LDA (20–100 μmol/L) but caused significant inhibition at higher concentrations: by 30% at 200 μmol/L and by 40% at 2000 μmol/L (Figure 2A). In agreement, LDA concentrations of salicylic acid had no effect on mRNA expression of either *sFlt1 i13* or *e15a*; however, a concentration-related inhibitory effect was observed at 200–2000 μmol/L (Figure 2B,C).

### 3.2. Effects of Aspirin and Salicylic Acid on Hypoxia-Induced sFlt1 Protein Release and mRNA Expression in BeWo Cells

**Figure 3:**
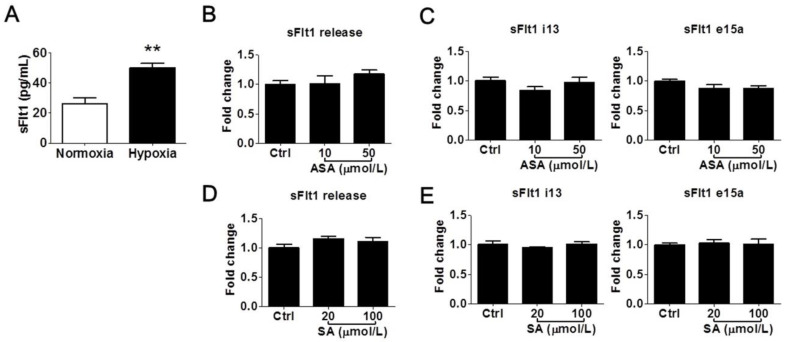
Low concentrations of aspirin (10 and 50 μmol/L) and salicylic acid (20 and 100 μmol/L) on hypoxia-induced sFlt1 protein release and mRNA expression in BeWo cells. (**A**) Hypoxia (1% oxygen, 24 h) induced sFlt1 release from BeWo cells. (**B**) Effect of aspirin on supernatant sFlt1 protein levels. (**C**) Effect of aspirin on *sFlt1* mRNA expression. (**D**) Effect of salicylic acid on supernatant sFlt1 protein concentrations. (**E**) Effect of salicylic acid on *sFlt1* mRNA expression. ASA, aspirin. SA, salicylic acid. Data are expressed as absolute values or fold changes relative to the control and presented as means ± SE, *n* = 4. ** *p* < 0.01.

We further determined whether LDA-relevant concentrations of aspirin and salicylic acid could modulate sFlt1 in BeWo cells under hypoxic conditions. As shown in Figure 3A, hypoxia treatment (1% oxygen for 24 h) increased supernatant sFlt1 protein concentrations in BeWo cells by approximately 2-fold vs. normoxia (50.0 ± 3.1 pg/mL vs. 26.3 ± 3.6 pg/mL, respectively). This effect was blocked by 10 nmol/L of the HIFα inhibitor chetomin [31]. We found that neither aspirin (10 and 50 μmol/L) nor salicylic acid (20 and 100 μmol/L) had any significant effect on sFlt1 protein release (Figure 3B,D) or mRNA expression (Figure 3C,E). We did not test higher drug concentrations in this hypoxia model, considering that they are not relevant to pregnant women.

### 3.3. Effects of Aspirin and Salicylic Acid on sFlt1 Protein Release and mRNA Expression in HTR-8/SVneo Cells

**Figure 4:**
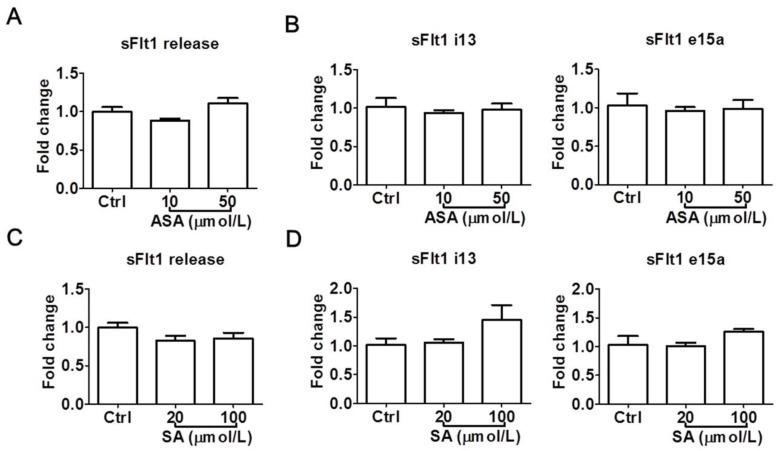
Low concentrations of aspirin (10 and 50 μmol/L) and salicylic acid (20 and 100 μmol/L) on sFlt1 protein release and mRNA expression in HTR-8/SVneo cells. (**A**,**B**) Effects of aspirin on supernatant sFlt1 protein levels and cellular mRNA expression. (**C**,**D**) Effects of salicylic acid on supernatant sFlt1 protein levels and cellular mRNA expression. ASA, aspirin. SA, salicylic acid. Data are expressed as fold changes relative to the control and presented as means ± SE, *n* = 4.

To confirm the above observations, we also examined the effects of aspirin and salicylic acid at LDA concentrations in a second trophoblast cell line, HTR-8/SVneo. Again, neither aspirin (10–50 μmol/L) nor salicylic acid (20–100 μmol/L) significantly reduced sFlt1 protein release (Figure 4A,C) or mRNA expression (Figure 4B,D). Salicylic acid at 100 μmol/L appeared to cause a non-significant increase of *sFlt1* mRNA expression, but not at the protein level. Since we previously found that sFlt1 protein release and mRNA expression were not stimulated by hypoxia in this cell line [31], we did not test drug effects under this condition.

## 4. Discussion

The clinical effects and mechanisms of action of aspirin vary with dosage. The recommended prophylactic LDA dosage for preeclampsia is typically 75–81 mg/day, although up to 150 mg/day has been used in some studies. The main established mechanism of action for LDA is irreversible acetylation of platelet cyclooxygenase (COX)-1, which results in reduced thromboxane A2 formation and a subsequent antithrombotic effect [41]. Aspirin also inhibits COX-2, although at a much lower potency, and this underlies its analgesic, antipyretic, and anti-inflammatory actions [42,43]. Aspirin has a unique pharmacokinetic–pharmacodynamic property: following oral administration, it is rapidly absorbed into the portal vein, where acetylation of platelet COX-1 occurs, and most of the parent drug is converted to salicylic acid by plasma and liver esterases before reaching the systemic circulation [44]. Thus, even though aspirin has a low plasma concentration (i.e., lower than that of salicylic acid) and a short plasma half-life of ~20 min, its antithrombotic action relies on circulating platelets (which have a lifespan of 8–12 days) with already inactivated COX-1, and not necessarily on systemic drug concentrations. However, we anticipate that mechanisms independent of platelets would be driven by plasma drug levels. With these considerations, we hypothesized that (1) if aspirin could directly modulate sFlt1 in trophoblasts, it must operate at relatively low concentrations consistent with the low doses used in pregnant women; (2) either aspirin or its major metabolite salicylic acid or both, may mediate the action; and (3) such an effect may occur via known or unknown mechanism(s).

A review of the clinical LDA data showed that plasma peak concentrations for aspirin and salicylic acid were in the range of single- and double-digit μmol/L, respectively (Table 1). While plasma concentrations of both aspirin and salicylic acid were dose-dependent, formulations were also important: the Cmax values were much lower for the modified-release formulations than the immediate-release formulations. This was particularly evident for plasma aspirin concentrations, as slow absorption of modified-release formulations favors the metabolic conversion of aspirin to salicylic acid in the portal circulation. Due to the short plasma half-lives of both aspirin and salicylic acid, there was no steady-state accumulation after chronic dosing.

In this study, we examined the effect of both aspirin and salicylic acid in two human trophoblast cell lines, BeWo and HTR-8/SVneo, simulating villous syncytiotrophoblasts and cytotrophoblasts, respectively. Our results show that both aspirin and salicylic acid indeed reduced sFlt1 protein release and mRNA expression, but only at near-millimolar to millimolar concentrations, and not at lower concentrations relevant to LDA. Furthermore, the LDA-relevant concentrations of both drugs failed to mitigate hypoxia-induced sFlt1 upregulation in BeWo cells. These data suggest that direct modulation of trophoblast release or expression of sFlt1 is unlikely to be the mechanism to account for aspirin’s clinical efficacy.

Our results are largely in line with the literature but with some exceptions. Overall, it appears that the earlier observations of a role for aspirin in reducing trophoblast sFlt1 were primarily from treatments at high concentrations. For example, Li et al. [19] found that aspirin reduced sFlt1 protein secretion and mRNA expression in human primary cytotrophoblasts and HTR-8/SVneo cells under both normoxia and hypoxia, but the effects were only seen at 3–12 mmol/L and not at lower concentrations. Su et al. [21] reported that aspirin was able to inhibit sFlt1 protein release from both human primary cytotrophoblasts and HTR-8/SVneo cells at 100 μmol/L and above, but they did not test lower concentrations. In contrast and consistent with our results, Han et al. [23] showed that aspirin at 10 μg/mL (55 μmol/L) did not modulate sFlt1 protein release from HTR-8/SVneo cells, either under basal conditions or in the presence of an antiphospholipid antibody. In a co-culture study of HTR-8/SVneo and endothelial cells, 100 μmol/L aspirin did not affect the protein or mRNA levels of either sFlt1 or VEGF [24]. While many earlier investigations focused on HTR-8/SVneo and primary cytotrophoblasts, only one has tested aspirin’s effect on sFlt1 in BeWo cells [20]. In that work, the authors observed that aspirin at 5 μg/mL (27.8 μmol/L) was able to suppress sFlt1 expression and release elicited by the serum from patients with preeclampsia. It is unclear if different experimental conditions might have attributed to the discrepancies between their findings and our present observation.

To date, only limited clinical studies have investigated the effect of LDA on circulating levels of sFlt1. Murtoniemi et al. found that 100 mg/day aspirin, initiated before 14 weeks of gestation, was associated with elevated plasma levels of PlGF at 26–28 weeks in high-risk pregnant women, but sFlt1 concentrations were not reported in their study [25]. Mayer-Pickel et al. showed that 75–150 mg/day aspirin overall did not affect the plasma sFlt1/PlGF ratio throughout gestation in women regardless of obstetric outcomes, although there appeared to be a transient reduction of the sFlt1/PlGF ratio at 11–14 weeks gestation, but not later, in a subset women with a pathologic first-trimester screening for preeclampsia risk [26]. Mone et al. recently evaluated the effect of aspirin (75 mg/day) on low-risk pregnant women and found no effect on serum biomarkers, including PlGF and pregnancy-associated plasma protein-A (PAPP-A); no sFlt1 data were reported in their study [27]. Thus, the available clinical evidence for a role of LDA in sFlt1 modulation is sparse and inconclusive, and further clinical biomarker investigation is needed to provide a definitive answer.

The strengths of our study include the examination of both aspirin and its metabolite salicylic acid at clinically relevant concentrations. We were able to confirm the findings of earlier studies that aspirin indeed inhibits sFlt1 secretion from trophoblasts but at high concentrations. More importantly, we showed that at lower concentrations relevant to LDA treatment in pregnant women, neither aspirin nor salicylic acid had any effect on sFlt1 under similar experimental conditions. To our knowledge, this report is the first to investigate the role of salicylic acid on sFlt1 expression in trophoblasts.

There are limitations and other considerations that apply to this work. Firstly, our findings were based on trophoblast cell lines, which may or may not reflect the properties of trophoblasts in vivo. Indeed, translational preeclampsia research is hindered by the lack of reliable models. Although primary cytotrophoblasts are considered the gold standard, they undergo rapid phenotypic switches once in culture and have a limited lifespan [45]. To circumvent translational difficulties, we have recently revisited and identified BeWo cells as a suitable surrogate model for preclinical studies, considering the sFlt1 response to pharmacologic agents [31]. It is also possible that single cell culture may not fully recapitulate the in vivo milieu, although our present data are in agreement with an earlier HTR-8/SVneo and endothelial cell co-culture study, which did not find aspirin (100 μmol/L) effective in modulating sFlt1 protein or mRNA levels [24]. Nevertheless, the many similarities of cellular responses to aspirin at low and high concentrations between our study and the published primary cytotrophoblasts lend support to the relevance of this investigation. Secondly, we used human plasma Cmax data to guide our experiments. Cmax occurs only transiently, and the average concentration over time is lower, whereas cultured cells were theoretically exposed to more constant drug levels. Although we did not determine the intracellular drug levels, the plasma compartment is considered to be equivalent to cell culture media for experimental purposes, and the lower protein concentration (~10% of human serum) in culture media theoretically favors the release of unbound, free drugs to the cells. Additionally, plasma exposures of both aspirin and salicylic acid are lower in pregnant than non-pregnant women due to hemodilution and altered pharmacokinetics [32,33], and the use of slow- rather than immediate-release formulations also reduces peak drug concentrations. Thus, we expect that the drug concentrations used in our experiments represent a conservative estimate of clinical scenarios; this notion is consistent with the earlier placental perfusion literature [46,47]. Thirdly, our present interpretation is limited to the direct effect of aspirin on trophoblast release/expression of sFlt1 but does not address other potential effects of aspirin and salicylic acid on trophoblasts (e.g., other pro- and anti-angiogenic factors such as PlGF and soluble endoglin), the downstream pathways of sFlt1, or the effects caused by global uteroplacental hemodynamic alterations.

## 5. Conclusions

The mechanism underlying the clinical efficacy of LDA in preeclampsia prevention remains not clearly understood. We examined whether aspirin and its principal metabolite, salicylic acid, might modulate sFlt1 release and/or expression in trophoblasts at clinically relevant concentrations. We found that in both normoxic and hypoxic conditions, neither aspirin nor salicylic acid modulated sFlt1 protein release or mRNA expression in cultured human trophoblasts at LDA-relevant concentrations. Further investigations are needed to understand whether aspirin and/or salicylic acid may act via alternate mechanisms and whether those might be utilized for the development of more potent and efficacious therapeutics for preeclampsia.

## Figures and Tables

**Table 1:** Systemic plasma exposures of aspirin and salicylic acid after oral low-dose aspirin in humans from published studies.

Aspirin Dose (mg/day or Single Dose)	Formulations	Aspirin Cmax (μmol/L)	Aspirin AUC (μmol/L∗h)	SA Cmax (μmol/L)	SA AUC (μmol/L∗h)	SA vs. Aspirin Cmax Ratio	Subjects	References
40	Immediate-release tablet	1.3 ± 0.4	2.1 ± 0.7	10.9 ± 2.1	44.9 ± 11.6	8.4	Healthy males and females	[35]
40	Extended-release tablet	0.2 ± 0.1	0.6 ± 0.5	3.8 ± 0.9	40.1 ± 13.4	18.1		
81	Immediate-release tablet	2.8 ± 0.8	4.8 ± 1.4	22.8 ± 4.9	101.4 ± 29.8	8.1		
81	Extended-release tablet	0.6 ± 0.3	1.8 ± 0.7	9.0 ± 3.5	87.6 ± 30.2	15.3		
50	Modified-release capsule	1.2 ± 0.6	2.1 ± 0.7	19.9 ± 2.5	10.6 ± 14.5	16.6	Healthy males and females	[36]
50	Solution	7.3 ± 4.1	3.8 ± 1.9	31.5 ± 3.7	78.2 ± 8.3	4.3		
75	Solution	7.3 ± 2.1	3.6 ± 0.5	~14.5	34.4 ± 8.8	2.0	Healthy males	[37]
75	Controlled-release tablet	0.5 ± 0.1	2.7 ± 0.6	4.9 ± 1.4	29.1 ± 8.0	9.2		
75	n/a	5.1 ± 2.6	4.5 ± 2.0	23.2 ± 5.9	54.8 ± 14.7	4.5	Healthy pregnant women (27–29 weeks)	[32]
75	n/a	5.1 ± 1.8	4.9 ± 2.0	17.8 ± 3.0	44.7 ± 8.5	3.5	Healthy pregnant women (36–38 weeks)	
75	n/a	7.4 ± 1.3	6.4 ± 2.0	29.6 ± 8.4	71.4 ± 24.3	4.0	Healthy males and non-pregnant females	
75	Controlled-release	0.3 ± 0.03	n/a	2.3 ± 0.4	n/a	7.9	Healthy males	[38]
162.5	Immediate-release	6.8 ± 1.3	n/a	15.4 ± 1.6	n/a	2.3		
80	Tablet	5.5 ± 1.3	5.2 ± 0.8	30.2 ± 6.7	88.5 ± 23.4	5.5	Healthy males	[39]
100	Tablet	5.6 ± 1.9	4.9 ± 2.2	30.3 ± 7.1	105.7 ± 28.0	5.4	Healthy males and females	[40]
100	n/a	23.4	n/a	57.4	n/a	2.5	Pregnant women	[34]
100	Enteric-coated tablet	n/a	n/a	9.2 ± 0.7	86.4 ± 0.8	n/a	High-risk pregnant women	[33]
100	Non enteric-coated tablet	n/a	n/a	17.5 ± 0.5	88.2 ± 0.7	n/a		
150	Non enteric-coated tablet	n/a	n/a	23.8 ± 0.8	144.8 ± 1.7	n/a		
100	Enteric-coated tablet	n/a	n/a	13.1 ± 1.1	159.4 ± 0.9	n/a	Non-pregnant women	
100	Non enteric-coated tablet	n/a	n/a	23.3 ± 0.7	152.8 ± 3.9	n/a		
150	Non enteric-coated tablet	n/a	n/a	34.7 ± 1.3	212.1 ± 3.1	n/a		

AUC, area under the curve from time 0 to 24 h or the last measurable concentration if before 24 h; Cmax, maximum drug concentration. Data include all oral aspirin formulations (solutions, immediate-release formulations, and modified-release formulations) and are expressed as means ± SD. Some of the originally published values were converted to the current units. SA, salicylic acid. n/a, not available. A 162.5 mg dose was also included as a close dose for reference.

## Data Availability

Data are contained within the article. The data supporting the findings of this study are available from the corresponding author upon reasonable request.

## Abbreviations

AUC	area under the curve
Cmax	maximum concentration
COX	cyclooxygenase
LDA	low-dose aspirin
PlGF	placental growth factor
sFlt1	soluble fms-like tyrosine kinase-1
VEGF	vascular endothelial growth factor
